# A comprehensive review of current practices, challenges, and future perspectives in Koi fish (*Cyprinus carpio* var. koi) cultivation

**DOI:** 10.14202/vetworld.2024.1846-1854

**Published:** 2024-08-24

**Authors:** Krisna Noli Andrian, Hevi Wihadmadyatami, Nastiti Wijayanti, Srikanth Karnati, Aris Haryanto

**Affiliations:** 1 Department of Biochemistry and Molecular Biology, Faculty of Veterinary Medicine, Universitas Gadjah Mada, Yogyakarta, Indonesia; 2 Department of Anatomy, Faculty of Veterinary Medicine, Universitas Gadjah Mada, Yogyakarta, Indonesia; 3 Department of Animal Physiology, Faculty of Biology, Universitas Gadjah Mada, Yogyakarta, Indonesia; 4 Institute of Anatomy and Cell Biology, Julius-Maximilians-Universität of Würzburg, Würzburg, Germany

**Keywords:** cultivation efficiency, *Cyprinus carpio* var. *koi*, sustainable management system

## Abstract

The Koi fish (*Cyprinus carpio* var. *koi*) is an ornamental fish with a high selling value because of its attractive colors, color patterns, body shape, and swimming motion. Koi fish is extensively traded in the international fish market because of their popularity among hobbyists from numerous countries worldwide. This review discusses various aspects of Koi fish cultivation, including genetic involvement, selective breeding strategies, and management systems. By examining crucial factors such as water parameters, technological innovations, and evolving cultivation methods, this review explored their influence on the quality of Koi fish. Breakthrough technologies, such as ornamental fish warehousing and recirculation aquaculture systems, enhance breeding efficiency and profitability. Molecular sexing, feed optimization, and color enhancement strategies are central to pursuing superior Koi fish. Reproduction management, disease prevention, and risk reduction during transport underscore ongoing efforts to ensure their survival. Despite notable progress, several challenges remain, including limited genetic studies, gaps in disease research, and unexplored herbal alternatives. The active involvement of hobbyists and breeders in research initiatives is a pivotal force in unlocking the untapped potential. The holistic approaches to enhance production efficiency and improve care standards require further exploration, paving the way for a sustainable future in the evolving management of Koi fish cultivation.

## Introduction

The Koi fish (*Cyprinus carpio* var. *koi*) is an ornamental fish that captivates several hobbyists because of its attractive colors, color patterns, body shape, and swimming patterns. Observing Koi fish is known to evoke a sense of peace. There are approximately 120 variants of Koi, the most commonly cultivated being Kohaku, Sanke, and Showa. These three groups are typically referred to as the Gosanke groups and are closely related [1, 2]. Japan exports ornamental fish, with Koi being the most widely exported species. The international market share for ornamental fish trade in Japan in 2008 was approximately 6.5% of the total world ornamental fish trade, amounting to approximately 21 million USD [3]. In 2022, Japan’s export of ornamental fish was approximately 48.6 million USD, growing more than twice in 14 years. The other top exporting countries for ornamental fish are Indonesia, with a trade value of 36.4 million USD, and Singapore, with 35.1 million USD. Koi fish is one of the most exportable species. The significant level of trade reflects the widespread interest in Koi fish among hobbyists worldwide, indicating its potential as a promising and growing business. The cultivation of Koi fish continues to evolve, with an increasing number of individuals recognizing the benefits of maintaining ornamental fish. Whether enjoyed in ponds or aquariums, the relaxation derived from observing these fish has become a significant aspect for hobbyists and holds the promise of a successful business for Koi fish breeders [4].

Koi fish rearing systems play a crucial role in their quality. Breeders make various efforts to enhance fish quality while minimizing production costs. This involves meticulous selection processes, whereby breeders select high-quality broodstocks and cull low-quality fish at a specific age to ensure the maintenance of superior Koi fish standards [1]. Another selection method involves molecular-assisted sexing the fish at a young age [5], enabling monosex cultivation specifically for male Koi, which are known for their tendency to exhibit more vibrant colors than females. The selection of Koi fish is crucial because of their inherent diversity, which ensures that they show various qualities even within a single lineage [2, 6].

The scarcity of published information on Koi fish, particularly in journals, necessitated an in-depth review. This review aims to fulfill the need for a comprehensive compilation, address current knowledge gaps, and serve as a valuable reference for those engaged in Koi fish-related studies and practices. This review describes various factors that influence the quality and survival of Koi fish. These aspects encompass topics such as fish-rearing management systems, enhancement of fish pigmentation, promotion of fish growth, and improvement in fish survival. This review additionally discusses the challenges and prospects of Koi fish management and research.

## Database Search

A systematic review of the Scopus-indexed electronic research databases was conducted on the various aspects of Koi fish. In addition, data from publications by international organizations, including the Food and Agriculture Organization and the World Bank, were used.

## A Comprehensive Approach to Koi Fish Cultivation

Fish cultivation management systems significantly influence fish cultivation. An intensive rearing system, coupled with regular assessments, can boost fish production trends, surpassing previously projected targets [7]. Monitoring the environment of Koi fish ensures their health and prevents their sudden death. Maintaining water quality by adhering to standards for parameters such as hydrogen power (pH), temperature, dissolved oxygen, salinity, ammonia (NH_3_), nitrate (NO_3_), nitrogen dioxide (NO_2_), iron (Fe), and phosphate (PO_4_) has proven to provide an optimal living environment for Koi fish [8, 9]. The other water quality parameters considered for fish cultivation are listed in Table-1 [10–16].

**Table-1:** Recommended water parameter criteria for fish.

Parameter	Acceptable range	Optimum range	Critical range	References
Temperature (°C)	15–35	23–25	Below 12 or above 35	[10-15]
Turbidity	20–120	30–80	Below 12 or above 120	[10, 14]
Watercolor	Pale to light green	Light green to light brown	Clearwater, dark green, and brown	[10, 14]
Dissolved oxygen (mg/L)	3–8	5–7	Below 5	[10, 13-15]
Biochemical oxygen demand (mg/L)	1–6	1–3	Above 10	[10, 14]
Carbon dioxide (mg/L)	0–10	Below 5 or 5–10	Above 12	[10, 14]
pH	7–9.5	7–7,5	Below 4 or Above 11	[10, 11, 13-15]
Alkalinity (mg/L)	25–200	25–100	Below 20 or above 300	[10, 13]
Hardness (mg/L)	Above 20	75–150	Below 20 or above 300	[10, 13]
Calcium (mg/L)	4–160	25–100	Below 10 or above 250	[10, 16]
Ammonia (mg/L)	0–0.05	0–0.025	Above 0.3	[10, 14]
Nitrite (mg/L)	0.02–2	Below 0.02	Above 0.2	[10, 14]
Nitrate (mg/L)	0–100	0.1–4.5	Below 0.01 or above 100	[10, 14]
Phosphorus (mg/L)	0.03–2	0.01–3	Above 3	[10, 14]
Hydrogen sulfide (mg/L)	0–0.02	0.002	Any detectable level	[10, 13]
Phytoplankton and zooplankton population (No. L^-1^)	2000–6000	3000–4500	Below 3000 or above 7000	[10]

Poor water quality can induce oxidative stress in fish, potentially leading to mortality [10]. Intensive monitoring of water quality is, therefore, essential to ensure optimal living conditions. In addition, contaminants such as microplastics [17] can adversely affect the growth and performance of the fish, underscoring the need for continuous monitoring of environmental pollutants. Recent advancements in the surveillance of fish water quality in aquatic environments have incorporated Internet of Things (IoT)-based tools. These recently developed tools demonstrate comprehensive operational efficiency and facilitate real-time and precise data transmission to satisfy specific production demands. Intelligent measurement equipment, specifically designed for continuous operation, significantly minimizes the losses attributed to personnel, material resources, and data errors. Technological innovation not only enhances operational efficiency but also offers substantial data and technical support for ongoing water quality regulation and management of aquaculture production [18–20].

Another factor to consider when maintaining Koi fish is the density of fish in ponds and aquariums. Fish density affects growth ratios, size variation, and mortality [21]. The denser the fish population in the pond, the lower the growth ratio, weight gain, and body length. There were also decreases in the specific growth rate, feed conversion ratio, feed efficiency ratio, and protein efficiency ratio [21, 22]. The recommended density of fish in an aquarium is 45 fish in a 150 L aquarium or 0.3 fish/L of water for young fish [23]. The recommended biofilter density is 1.4 kg/m^3^ when maintained in an aquaponica system enriched with spinach (*Beta vulgaris* var. *bengalensis*) [21] and 2.1 kg/m^3^ in that enriched with gotukola (*Centella asiatica*) [24]. However, economic factors related to fish density should be considered. Lower fish densities are economically less efficient because the number of fish that can be sold during cultivation decreases. It is recommended that high-density ponds frequently change approximately 20% of the total water content every day to increase the survivability of fish at a young age [25]. Survival rates also decrease at high fish densities because of competition for food and stress caused by reduced space for fish movement [21].

Several Koi fish are maintained in ponds and aquariums. Rearing them in ponds with muddy bottoms tends to be a better choice than in aquariums because of their assimilation capacity and the presence of more plankton [26]. The system for cultivating Koi fish has evolved from the traditional method of maintaining Koi, in ponds or aquariums to the contemporary approach of developing aquaponic systems [16, 21, 22, 24, 27–32]. Aquaponic systems not only help remove pollutants produced by fish, thereby reducing water waste levels but also allow for the simultaneous cultivation of fish and plants [21]. The plants commonly used in aquaponic systems for Koi fish are listed in Table-2 [16, 21, 22, 24, 27–32].

**Table-2:** List of plants used for aquaponic system in Koi fish culture.

Species	Common name	Optimum recommendation	References
*Beta vulgaris var. bengalensis*	Spinach beet	1.4 kg/m^3^ stocking density	[21]
*Ipomoea aquatica*	Water spinach	0.8 kg/m^3^ stocking density dan 28 plant/m^2^	[22]
*Centella asiatica*	Gotukola	2.1 kg/m^3^ stocking density	[16, 24, 29]
*Lactuca sativa*	Lettuce	10.7 kg/m^3^ stocking density	[27]
*Eichhornia crassipes*	Hyacinth	4 bushes of hyacinth per tank	[28]
*Ceratophyllum demersum*	Coontail	400 fish in 10 m^3^ water with 12 connected mini pond	[30]
*Utticularia* spp.	Bladderwort	400 fish in 10 m^3^ water with 12 connected mini pond	[30]
*Salvinia* spp.	Salvinia	400 fish in 10 m^3^ water with 12 connected mini pond	[30]
*Pygmaea helvola*	Hardy waterlily	400 fish in 10 m^3^ water with 12 connected mini pond	[30]
*Lythrum salicaria*	Purple loosestrife	400 fish in 10 m^3^ water with 12 connected mini pond	[30]
*Azolla* spp.	Mosquito fern	400 fish in 10 m^3^ water with 12 connected mini pond	[30]
*Carex riparia*	Greater pond sedge	400 fish in 10 m^3^ water with 12 connected mini pond	[30]
*Pontederia cordata*	Pickerelweed	400 fish in 10 m^3^ water with 12 connected mini pond	[30]
*Cyperus alternifolius*	Umbrella sedge	400 fish in 10 m^3^ water with 12 connected mini pond	[30]
*Lactuca sativa* var. *Crispa*	Leaf lettuce	5 kg/m^3^ stocking density	[31]
*Fragaria* spp.	Strawberry	5 kg/m^3^ stocking density	[31]
*Hydrocotyle rotundifolia*	Lawn marsh pennywort	1.5 kg in 200 L water	[32]

Another breakthrough in maintenance systems has been the increasingly advanced use of technology and big data. One notable example is the ornamental fish warehousing (OFWare) system [33], developed to assist breeders in various aspects of the Koi fish business. This system aids in calculating business aspects, projecting trait inheritance, implementing tagging for ownership and origin, organizing system-based competition, and employing anti-fish theft mechanisms by integrating big data computed from fish photos and videos. Developed using machine learning, OFWare simplifies Koi fish breeding and boosts breeder profits. Another management system under development is the recirculation aquaculture system (RAS) [34], which was designed to enhance the profitability of fish farming. The RAS provides maximum profit projections, with simulations indicating potential profits up to 10 times. The influential factors include the timing of introducing a batch of fish, initial fish size, growth ratio, and pre-calculated sales factors [34]. The RAS system utilizes reused water and employs filters made from materials such as zeolite (bear blanked clinoptilolite, mordenite manganese, and geopolymeric zeolite A [35]) and biofilters in aquaponic systems [16, 21, 22, 24, 27–32]. These breakthroughs and innovations in fish farming, which involve monitoring with IoT-based tools, implementing aquaponic systems, utilizing OFWare, and adopting RAS systems, aim to improve fish quality, enhance overall living conditions, promote growth, and increase breeder profits. Selective breeding of Koi fish is strictly performed to ensure their quality as they mature, thereby reducing operational costs. Fish selection currently involves selecting a good broodstock [1, 8] and assessing the phenotype of the offspring, which are periodically culled [1]. The culling process occurs in stages for Koi juveniles, with variants such as Kohaku experiencing 60%–65% culling at 40 days of age, Sanke undergoing 75%–80% culling at 25–30 days of age, and Showa facing 50%–95% culling at 3–5 days of age. Variants with a single color also undergo a strict culling process based on quality. This selection process continues until the fish are 6 months old, ensuring that only high-quality fish remain. Evaluation of Koi fish quality focuses on body conformation, swimming style, color quality, color durability, and color distribution [1]. The price of the Koi fish was determined based on the results of this evaluation, particularly if the fish had a record of winning a Koi fish contest. Selective breeding enhances the efficiency of fish-rearing management, allowing breeders to obtain high-quality fish at low operational costs [1, 34]. Selective breeding using molecular markers provides an alternative to mono-sex culture because male Koi tend to have more vibrant colors than female Koi. Determining sex before the Koi reaches 5 months or approximately 20–30 cm in size is challenging. The use of ArS.9–15 primers, which target sex-specific markers, for polymerase chain reaction (PCR) amplification has proven effective in differentiating between male and female Koi [5]. It is hoped that breeders will apply this breakthrough technique to determine the sex of Koi fish to maintain a mono-sex culture from an early age, thereby achieving cost-efficiency in producing fish of the highest quality.

**Figure-1:**
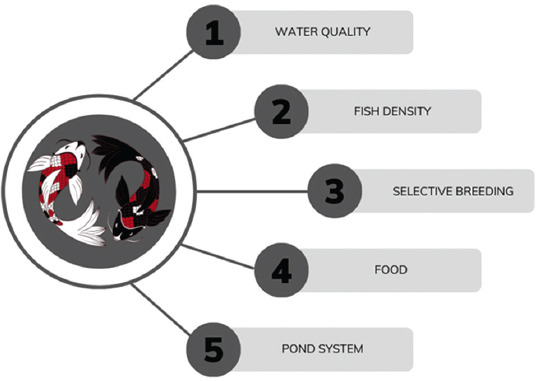
Vital points in Koi fish management.

Another crucial aspect of the maintenance system that needs consideration is the food provided to Koi fish. The effect of feed was positive when administered at specific proportions. Presently, Koi fish feed is widely available and is mass-produced by manufacturers or derived from natural sources, including mixtures of various ingredients [36]. Natural foods commonly provided to Koi fish include spirulina (*Arthrospira maxima*) [37, 38], tubifex (*Tubifex tubifex*), and zooplankton [39]. The typical macromolecular composition of Koi feed used for fish maintenance is 31%–33% protein, 4%–6% fat, 3%–5% fiber, and 9%–10% moisture content, with recommended feeding twice a day [8]. Overfeeding can lead to water turbidity and excessive ammonia production, thereby deteriorating the water quality and causing oxidative stress in fish [10]. Specific feed treatments and their effects are discussed in the next section, including aspects such as pigmentation enhancement, growth rate, reproduction, survivability, and immunomodulation. A summary of the vital management points is presented in Figure-1.

## Koi Fish Coloration: Genetic Factors and Enhancement Strategies

The color variation of Koi fish is a primary attraction for hobbyists because of the appealing patterns. Koi fish boast a variety of colors, including white, red, black, yellow, silver, orange, and blue, creating attractive color compositions based on Koi variants. Koi variants can exhibit a solid monochromatic color or a mix of two or three colors, thereby distinguishing them from other variants. Breeders and researchers have made various efforts to reveal insights into the color and pigmentation of Koi fish. The patterns and pigmentation of fish originate from specific combinations of chromatophores. Several hormones, such as melanin-concentrating hormone, melanocyte-stimulating hormone, agouti signaling protein, and thyroid hormone, play their roles in regulating pigmentation in fish [40]. The genes reported to play a major role in pigmentation in Koi fish are melanocortin 1 receptor and microphthalmia-associated transcription factor A, which serve as regulators of pigment development in Koi fish [41, 42]. Various micro RNA (miRNAs) have been reported to influence the regulation of pigmentation in Koi fish, including miRNA-430b [43], miRNA-137 [44], miRNA-206 [45], and miRNA-196a [46], as well as numerous other miRNAs that have not yet been extensively studied [47]. Long non-coding RNAs have also been reported to play complex roles in the regulation of pigmentation in Koi fish [48, 49]. The dynamic regulation by mRNA and miRNAs at various stages of fish development indicates that numerous regulators influence pigmentation in Koi fish [50, 51]. Several other factors can affect the pigmentation process in fish, including environmental conditions, the type of food provided, illumination of the fish, interactions with other fish, and the influence of inherited genetic factors [40, 52–54].

Breeders attempt to enhance the color quality of Koi fish by providing food that increases its sharpness. The price of fish tends to be higher when the colors are vibrant, which makes them more appealing. Commercial feeds are often advertised for their ability to enhance color sharpness by incorporating proven ingredients. These feeds typically contain additives such as algae, crustaceans, β-carotene, canthaxanthin, zeaxanthin, and astaxanthin [55]. Supplements such as Carophyll^®^ Red (DSM Nutritional Products Ltd. Netherlands) (synthetic carotenoid), *Rhodopseudomonas palustris* (photosynthetic bacteria), and *Spirulina platensis* have been proven effective in enhancing the color of the Showa variant [38]. Reports suggest that adding pumpkin meal to Koi fish feed (consisting of 20% of the total feed) can enhance the color of the Kohaku variant [56]. Similarly, adding 10% red dragon fruit peel meal to fish feed positively affected the color quality of Koi due to its astaxanthin content [57]. The addition of 250 mg of astacin to 1 kg of fish feed has also been reported to positively affect the color of Koi fish [58]. Additional supplements such as *Chlorella vulgaris, Haematococcus pluvialis*, and *A. maxima* have been reported to be effective in improving color quality [37]. The numerous available supplement options allow breeders to choose from a variety of alternatives. However, research on more cost-effective materials for enhancing Koi fish color should continue to provide alternatives and reduce production costs with optimal results.

## Enhancing Koi Fish Growth: Key Supplements and Systems

In addition to their appealing color patterns, the market value of Koi fish has increased in size and proportion. Several Koi fish opportunists seek seeds with rapid growth potential for efficient cultivation. Various studies have recommended feeds and supplements, such as selenium (Se) supplementation in aquaponic systems, to accelerate the growth of Koi fish [27]. The administration of selenium (Se) supplements at levels ranging from 1.55 to 1.57 mg/kg has been demonstrated to have a positive impact on Koi fish growth. Another nanoparticle, iron oxide (N_2_O_3_), when added to fish feed at a concentration of 30 mg/100 g, demonstrated an efficiency in the relationship between the quantity of feed provided and fish growth [59]. Supplementing additional feed, like *Arthrospira platensis*, is reported to enhance growth, pigmentation, and digestive system activity in fish, with 5%–10% additional *A. platensis* has been proven effective when added to fish feed [60]. Providing *Artemia nauplii* in the form of decapsulate cysts has also been reported to promote growth in Koi fish by increasing the activity of digestive system enzymes, including protease, amylase, chitinase, and lipase [61]. Alternative feeds for juvenile Koi fish, such as soybean meal or hazelnut meal, can be safely administered either by mixing with other feeds or provided as standalone options without adverse effects on fish [36]. The administration of a chitosan nanoemulsion mixed with *Chlorella* enhanced the growth of Koi fish, accompanied by improvements in water quality within the cultivation system and enhanced protein retention [62]. Implementing a high-energy/protein feeding system for 2 weeks, followed by low-energy/protein feeding, has been shown to increase fish production efficiency without negatively affecting Koi fish biomass. This system positively affected fish nutrition by enhancing the nutritional composition, energy density, and biomass production of forage Koi [63]. Aquaculture systems, such as aquaponics, have also proven beneficial in enhancing the quality of life and growth of Koi fish. For example, the addition of Gotukola (*C. asiatica* [L.]) to aquaponic systems was demonstrated to increase fish growth and improve water quality [16, 24]. With the availability of various supplements and alternative feeds with proven beneficial effects on fish growth, Koi fish breeders and hobbyists can use these supplements as references for effective cultivation, ultimately boosting the growth of the fish raised. A summary of the various supplements and systems used for Koi fish cultivation is presented in Table-3 [16, 24, 36, 62, 63].

**Table-3:** Summary of supplements and systems for Koi fish cultivation.

Supplement/System	Effect	Amount given/System	References
Aquaponics with Gotukola addition	Improves quality of life, growth, and water quality	In an aquaponic system, Gotukola is planted with a density of 35/m^2^ and fish are stocked at a density of 2.1 kg/m^3^	[16, 24]
Se supplementation	Positive impact on Koi fish growth	1.55–1.57 mg/kg	[27]
Soybean meal or hazelnut meal	Safe alternative feed for juvenile Koi fish	50%–100% of total feed given	[36]
Iron oxide (N_2_O_3_) supplementation	Efficient growth in relation to feed quantity	30 mg/100 g	[59]
*Arthrospira platensis*	Enhances growth, pigmentation, and digestive activity	5%–10% additional to feed	[60]
*Artemia nauplii* in decapsulated cysts form	Promotes growth by increasing digestive enzyme activity in juvenile	10% of the total biomass of the juvenile	[61]
Chitosan nanoemulsion	Enhances growth, improves water quality, and protein retention	40% additional to feed	[62]
High-Energy to Low-Energy feeding system	Increases fish production efficiency without negative effects	Two weeks of high-energy feed, followed by 4 weeks of low-energy feed	[63]

## Optimizing Koi Fish Reproduction

Selecting broodstock is crucial for enhancing Koi fish reproduction. Broodstock selection should be based on color quality, body weight, length, and age. This approach aims to ensure that high-quality parents pass on desirable phenotypic characteristics to their offspring, ultimately producing high-quality progeny [1, 8, 64]. Male fertility is checked before spawning by squeezing the abdomen to release, milky-white sperm. Similarly, female fertility is assessed by examining the eggs produced by abdominal squeezing, producing yellow-colored eggs [8]. The recommended male-to-female ratio is typically 3:2 to maximize the chances of egg fertilization [64]. A higher ratio of males to females has been reported to yield greater hatching rates, reaching up to 80% [8] and 71% [64]. In addition, the presence of *Hydrilla* spp. in ponds can influence the fertility and hatching rates of Koi fish [64].

Research on Koi fish reproduction has exhibited a notable gap in terms of extensive exploration and documentation. Despite the critical importance of successful reproduction in sustaining and advancing Koi fish populations, few comprehensive studies are available in this domain. Key aspects, such as optimal breeding conditions, genetic influences on reproductive traits, and innovative techniques for enhancing reproductive efficiency, remain to be areas of limited research. Although there is not an extensive body of literature on increasing Koi fish reproduction, these practices can serve as valuable references for enhancing fish cultivation.

## Strategies for Enhancing Koi Survival and Immunomodulation

Koi fish cultivation is closely linked to the risk of fish mortality due to diseases or mishandling during cultivation and transfer. This concern has prompted breeders to take preventive measures against diseases and mistreatment that could endanger the lives of the Koi. Reports have highlighted the detrimental impact of parasites on fish growth, leading to efforts to use essential oils from *Lippia* spp. as anti-parasitic agents [65]. Spore proteins from *Myxobolus Koi* and red ginger have been explored as antihelminthic agents for controlling infections in Koi fish [66, 67]. Alkaloids derived from *Sanguinaria canadensis* roots, specifically sanguinarine (SAG), have anti-inflammatory and antioxidant effects, enhancing non-specific immunity and defensive responses against *Aeromonas hydrophila* infection in Koi fish [68]. Incorporating eryngii mushroom (*Pleurotus eryngii*) powder into fish feed has proven beneficial for Koi fish by boosting the innate immune response, enhancing mucus bactericidal activity on fish skin, and improving overall growth performance [69]. Beyond disease prevention and treatment, the process of transferring or transporting Koi fish carries risks due to factors such as vibration, temperature fluctuations, and oxygen levels. These factors, particularly those that induce oxidative stress, can cause fish mortality during transportation. It is, therefore, crucial to employ appropriate techniques and conditions to minimize risk during transportation and ensure the well-being of fish [70]. This may involve the use of commonly used sedatives in fish, such as MS-222 and propofol [71], to mitigate the impact of transportation on Koi fish. One noteworthy alternative is the I-Tiao-gung (*Glycine tomentella*) extract, known for its ability to maintain oxidative stress levels in Koi fish during transportation [72]. These diverse efforts, which encompass both prevention and handling strategies, are crucial for ensuring the well-being of Koi, preventing losses to breeders, and preserving cherished fish for hobbyists.

Substantial research gaps persist in enhancing the survival and immunomodulatory effects of Koi fish. There is a clear need for a more comprehensive exploration of strategies and interventions to enhance the survival rate of Koi and effectively modulate immune responses. Despite the promising nature of this field, the use of herbal alternatives has not been extensively studied. Despite advancements in various aspects of Koi fish care, specific attention to these areas is crucial for optimizing Koi health and resilience.

## Challenges and Future Prospects

The cultivation of Koi fish poses various challenges in achieving high-quality yields and efficient production costs. Despite the extensive literature on Koi fish rearing systems, including aspects such as management, fish selection, feeding, and treatments for pigmentation, growth, reproduction, and survival, certain critical areas require further investigation. At present, there are significant gaps in Koi fish research, with the following challenges:

1. Genetic studies: Limited research on the genetics of Koi fish, despite their large number of variants, hinders their potential for targeted improvements in traits.
2. Disease research: Despite their significant impact on mortality, the scarcity of in-depth studies on Koi fish diseases presents a crucial research gap.
3. Genetically based selection: Minimal application of genetically based selection methods in Koi fish, in contrast to other fish species.
4. Herbal alternatives: The lack of research on herbal alternatives for immunomodulation in Koi fish represents an unexplored area of health enhancement.
5. Impact of International Trade: The rising trend in the international Koi fish trade requires comprehensive studies on the potential challenges and impacts of crossbreeding between fish from different regions.
6. Role of hobbyists and breeders: Active engagement of hobbyists and breeders in research initiatives is crucial for uncovering untapped sectors in Koi fish cultivation.
7. Holistic management efficiency approaches: Research gaps exist in exploring holistic approaches to enhance the overall production efficiency and care standards in Koi fish farming.

Addressing these research gaps will not only contribute to the current understanding of Koi fish species but also foster advances in breeding practices, disease management, and sustainable international trade.

## Conclusion

Koi fish hold significant ornamental value and economic importance due to its attractive appearance and global demand. This comprehensive review has highlighted the multifaceted aspects of Koi fish cultivation, including genetics, disease management, and selective breeding. The integration of advanced technologies such as OFWare and RAS has the potential to revolutionize breeding practices and enhance profitability. Furthermore, molecular sexing, optimized feeding strategies, and color enhancement techniques are pivotal for producing superior Koi fish. Despite advancements, challenges such as limited genetic research, disease studies, and the potential of herbal alternatives remain. The engagement of hobbyists and breeders in research is essential for further progress. This review underscores the need for continuous exploration and holistic approaches to improve production efficiency and care standards, ensuring a sustainable future for Koi fish cultivation.

## Authors’ Contributions

All authors contributed to the conception and design of this review article. KNA: Conducted the article collection and drafting. HW, NW, SK, and AH: Reviewed the draft and provided feedback to improve the manuscript. All authors have read, reviewed, and approved the final manuscript.
